# Executive Summary: A Compendium of Strategies to Prevent Healthcare-Associated Infections in Acute-Care Hospitals: 2022 Updates

**DOI:** 10.1017/ice.2023.138

**Published:** 2023-10

**Authors:** Deborah S. Yokoe, Sonali D. Advani, Deverick J. Anderson, Hilary M. Babcock, Michael Bell, Sean M. Berenholtz, Kristina A. Bryant, Niccolò Buetti, Michael S. Calderwood, David P. Calfee, Erik R. Dubberke, Katherine D. Ellingson, Neil O. Fishman, Dale N. Gerding, Janet Glowicz, Mary K. Hayden, Keith S. Kaye, Michael Klompas, Larry K. Kociolek, Emily Landon, Elaine L. Larson, Anurag N. Malani, Jonas Marschall, Jennifer Meddings, Leonard A. Mermel, Payal K. Patel, Trish M. Perl, Kyle J. Popovich, Joshua K. Schaffzin, Edward Septimus, Kavita K. Trivedi, Robert A. Weinstein, Lisa L. Maragakis

**Affiliations:** 1 University of California San Francisco School of Medicine, UCSF Health-UCSF Medical Center, San Francisco, California, United States; 2 Duke University School of Medicine, Durham, North Carolina, United States; 3 BJC Healthcare, Washington University School of Medicine, St. Louis, Missouri, United States; 4 Centers for Disease Control and Prevention, Atlanta, Georgia, United States; 5 Johns Hopkins University, Baltimore, Maryland, United States; 6 Norton Healthcare, University of Louisville School of Medicine, Louisville, Kentucky, United States; 7 Infection Control Programme, Geneva University Hospitals and Faculty of Medicine, World Health Organization Collaborating Center, Geneva, Switzerland; 8 IAME-U1137, Université Paris-Cité, INSERM, Paris, France; 9 Dartmouth Hitchcock Medical Center, Lebanon, New Hampshire, United States; 10 Weill Cornell Medicine, New York, New York, United States; 11 Washington University School of Medicine, St. Louis, Missouri, United States; 12 College of Public Health, The University of Arizona, Tucson, Arizona, United States; 13 Penn Medicine, Hospital of the University of Pennsylvania, Philadelphia, Pennsylvania, United States; 14 Edward Hines Jr. Veterans’ Affairs Hospital, Hines, Illinois, United States; 15 Rush University Medical Center, Chicago, Illinois, United States; 16 Rutgers Robert Wood Johnson Medical School, New Brunswick, New Jersey, United States; 17 Harvard Medical School, Brigham and Women’s Hospital, Boston, Massachusetts, United States; 18 Northwestern University Feinberg School of Medicine, Ann & Robert H. Lurie Children’s Hospital of Chicago, Chicago, Illinois, United States; 19 The University of Chicago Medical Center, MacLean Center for Clinical Medical Ethics, Chicago, Illinois, United States; 20 Columbia School of Nursing, New York, New York, United States; 21 Trinity Health Michigan, Ann Arbor, Michigan, United States; 22 Bern University Hospital, University of Bern, Bern, Switzerland; 23 University of Michigan Medical School, Ann Arbor, Michigan, United States; 24 Veterans’ Affairs Ann Arbor Healthcare System, Ann Arbor, Michigan, United States; 25 Warren Alpert Medical School of Brown University, Providence, Rhode Island, United States; 26 Lifespan Hospital System, Providence, Rhode Island, United States; 27 Intermountain Healthcare, Salt Lake City, Utah, United States; 28 University of Texas Southwestern Medical Center, Dallas, Texas, United States; 29 Children’s Hospital of Eastern Ontario, University of Ottawa, Ottawa, Ontario, Canada; 30 Texas A&M College of Medicine, Houston, Texas, United States; 31 Harvard Pilgrim Healthcare, Boston, Massachusetts, United States; 32 Alameda County Public Health Department, San Leandro, California, United States; 33 Cook County Health, Chicago, Illinois, United States; 34 Johns Hopkins University School of Medicine, The Johns Hopkins Hospital, Baltimore, Maryland, United States

## Introduction to the Executive Summary

This document summarizes the recommendations that are included within each section of “A Compendium of Strategies to Prevent Healthcare-Associated Infections in Acute-Care Hospitals: 2022 Updates.”^1–8^

Appendix Tables 1 and 2 describe the criteria used to determine the quality of evidence ratings and classification of recommendations as essential practices versus additional approaches. These criteria are discussed in more detail in the “Introduction to a Compendium of Strategies to Prevent Healthcare-Associated Infections in Acute-Care Hospitals: 2022 Updates,”^9^ which also provides a summary of the background and the methods used to develop the *Compendium: 2022 Updates*. The individual *Compendium 2022* sections^1–8^ summarize the major changes to the recommendations from the *Compendium: 2014 Updates*.^10^

Appendix Table 3 lists the authors and members of the Advisory Group and Expert Panel for the *Compendium: 2022 Updates*.

## Executive summary

### Strategies to prevent catheter-associated urinary tract infections (CAUTIs)

**Table:** 

Essential practices	
**Infrastructure and resources**	
1	Perform a CAUTI risk assessment and implement an organization-wide program to identify and remove catheters that are no longer necessary using 1 or more methods documented to be effective. (Quality of evidence: MODERATE)
2	Provide appropriate infrastructure for preventing CAUTI. (Quality of evidence: LOW)
3	Provide and implement evidence-based protocols to address multiple steps of the urinary catheter life cycle: catheter appropriateness (step 0), insertion technique (step 1), maintenance care (step 2), and prompt removal (step 3) when no longer appropriate. (Quality of evidence: LOW)
4	Ensure that only trained healthcare personnel (HCP) insert urinary catheters and that competency is assessed regularly. (Quality of evidence: LOW)
5	Ensure that supplies necessary for aseptic technique for catheter insertion are available and conveniently located. (Quality of evidence: LOW)
6	Implement a system for documenting the following in the patient record: physician order for catheter placement, indications for catheter insertion, date and time of catheter insertion, name of individual who inserted catheter, nursing documentation of placement, daily presence of a catheter and maintenance care tasks, and date and time of catheter removal. Record criteria for removal and justification for continued use. (Quality of evidence: LOW)
7	Ensure that sufficiently trained HCP and technology resources are available to support surveillance for catheter use and outcomes. (Quality of evidence: LOW)
8	Perform surveillance for CAUTI if indicated based on facility risk assessment or regulatory requirements. (Quality of evidence: LOW)
9	Standardize urine culturing by adapting an institutional protocol for appropriate indications for urine cultures in patients with and without indwelling catheters. Consider incorporating these indications into the electronic medical record, and review indications for ordering urine cultures in the CAUTI risk assessment. (Quality of evidence: LOW)
**Education and training**	
1	Educate HCP involved in the insertion, care, and maintenance of urinary catheters about CAUTI prevention, including alternatives to indwelling catheters, and procedures for catheter insertion, management, and removal. (Quality of evidence: LOW)
2	Assess healthcare professional competency in catheter use, catheter care, and maintenance. (Quality of evidence: LOW)
3	Educate HCP about the importance of urine-culture stewardship and provide indications for urine cultures. (Quality of evidence: LOW)
4	Provide training on appropriate collection of urine. Specimens should be collected and should arrive at the microbiology laboratory as soon as possible, preferably within an hour. If delay in transport to the laboratory is expected, samples should be refrigerated (no more than 24 hours) or collected in preservative urine transport tubes. (Quality of evidence: LOW)
5	Train clinicians to consider other methods for bladder management, such as intermittent catheterization or external male or female collection devices, when appropriate, before placing an indwelling urethral catheter. (Quality of evidence: LOW)
6	Share data in a timely fashion and report to appropriate stakeholders. (Quality of evidence: LOW)
**Insertion of indwelling catheters**	
1	Insert urinary catheters only when necessary for patient care and leave in place only as long as indications remain. (Quality of evidence: MODERATE)
2	Consider other methods for bladder management such as intermittent catheterization, or external male or female collection devices, when appropriate. (Quality of evidence: LOW)
3	Use appropriate technique for catheter insertion. (Quality of evidence: MODERATE).
4	Consider working in pairs to help perform patient positioning and monitor for potential contamination during placement. (Quality of evidence: LOW)
5	Practice hand hygiene (based on CDC or WHO guidelines) immediately before insertion of the catheter and before and after any manipulation of the catheter site or apparatus. (Quality of evidence: LOW)
6	Insert catheters following aseptic technique and using sterile equipment. (Quality of evidence: LOW)
7	Use sterile gloves, drape, and sponges, a sterile antiseptic solution for cleaning the urethral meatus, and a sterile single-use packet of lubricant jelly for insertion. (Quality of evidence: LOW)
8	Use a catheter with the smallest feasible diameter consistent with proper drainage to minimize urethral trauma but consider other catheter types and sizes when warranted for patients with anticipated difficult catheterization to reduce the likelihood that a patient will experience multiple, sometimes traumatic, catheterization attempts. (Quality of evidence: LOW)
**Management of indwelling catheters**	
1	Properly secure indwelling catheters after insertion to prevent movement and urethral traction. (Quality of evidence: LOW)
2	Maintain a sterile, continuously closed drainage system. (Quality of evidence: LOW)
3	Replace the catheter and the collecting system using aseptic technique when breaks in aseptic technique, disconnection, or leakage occur. (Quality of evidence: LOW)
4	For examination of fresh urine, collect a small sample by aspirating urine from the needleless sampling port with a sterile syringe/cannula adaptor after cleansing the port with disinfectant. (Quality of evidence: LOW)
5	Facilitate timely transport of urine samples to laboratory. If timely transport is not feasible, consider refrigerating urine samples or using sample-collection cups with preservatives. Obtain larger volumes of urine for special analyses (eg, 24-hour urine) aseptically from the drainage bag. (Quality of evidence: LOW)
6	Maintain unobstructed urine flow. (Quality of evidence: LOW)
7	Employ routine hygiene. Cleaning the meatal area with antiseptic solutions is an unresolved issue, though emerging literature supports chlorhexidine use prior to catheter insertion. Alcohol-based products should be avoided given concerns about the alcohol causing drying of the mucosal tissues. (Quality of evidence: LOW)

Additional approaches	
1	Develop a protocol for standardizing diagnosis and management of postoperative urinary retention, including nurse-directed use of intermittent catheterization and use of bladder scanners when appropriate as alternatives to indwelling urethral catheterization. (Quality of evidence: MODERATE)
2	Establish a system for analyzing and reporting data on catheter use and adverse events from catheter use. (Quality of evidence: LOW)
3	Establish a system for defining, analyzing, and reporting data on non–catheter-associated UTIs, particularly UTIs associated with the use of devices being used as alternatives to indwelling urethral catheters. (Quality of evidence: LOW)

### Strategies to prevent central-line–associated bloodstream infections (CLABSIs)

**Table:** 

Essential practices	
**Before insertion**	
1	Provide easy access to an evidence-based list of indications for CVC use to minimize unnecessary CVC placement. (Quality of evidence: LOW)
2	Require education and competency assessment of healthcare personnel (HCP) involved in insertion, care and maintenance of CVCs about CLABSI prevention. (Quality of evidence: MODERATE)
3	Bathe ICU patients aged >2 months with a chlorhexidine preparation on a daily basis. (Quality of evidence: HIGH)
**At insertion**	
1	In ICU and non-ICU settings, a facility should have a process in place, such as a checklist, to ensure adherence to infection prevention practices at the time of CVC insertion. (Quality of evidence: MODERATE)
2	Perform hand hygiene prior to catheter insertion or manipulation. (Quality of evidence: MODERATE)
3	The subclavian site is preferred to reduce infectious complications when the catheter is placed in the ICU setting. (Quality of evidence: HIGH)
4	Use an all-inclusive catheter cart or kit. (Quality of evidence: MODERATE)
5	Use ultrasound guidance for catheter insertion. (Quality of evidence: HIGH)
6	Use maximum sterile barrier precautions during CVC insertion. (Quality of evidence: MODERATE)
**After insertion**	
1	Ensure appropriate nurse-to-patient ratio and limit use of float nurses in ICUs. (Quality of evidence: HIGH)
2	Use chlorhexidine-containing dressings for CVCs in patients aged >2 months. (Quality of evidence: HIGH)
3	For nontunneled CVCs in adults and children, change transparent dressings and perform site care with a chlorhexidine-based antiseptic at least every 7 days or immediately if the dressing is soiled, loose, or damp. Change gauze dressings every 2 days or earlier if the dressing is soiled, loose, or damp. (Quality of evidence: MODERATE)
4	Disinfect catheter hubs, needleless connectors, and injection ports before accessing the catheter. (Quality of evidence: MODERATE)
5	Remove nonessential catheters. (Quality of evidence: MODERATE)
6	Routine replacement of administration sets not used for blood, blood products, or lipid formulations can be performed at intervals up to 7 days. (Quality of evidence: HIGH)
7	Perform surveillance for CLABSI in ICU and non-ICU settings. (Quality of evidence: HIGH)

Additional approaches	
1	Use antiseptic or antimicrobial-impregnated CVCs. (Quality of evidence: HIGH in adult patients; MODERATE in pediatric patients)
2	Use antimicrobial lock therapy for long-term CVCs. (Quality of evidence: HIGH)
3	Use recombinant tissue plasminogen activating factor (rt-PA) once weekly after hemodialysis in patients undergoing hemodialysis through a CVC. (Quality of evidence: HIGH)
4	Utilize infusion or vascular access teams for reducing CLABSI rates. (Quality of evidence: LOW)
5	Use antimicrobial ointments for hemodialysis catheter-insertion sites. (Quality of evidence: HIGH)
6	Use an antiseptic-containing hub, connector cap, or port protector to cover connectors. (Quality of evidence: MODERATE)

### Strategies to prevent Clostridioides difficile infections (CDIs)

**Table:** 

Essential practices	
1	Encourage appropriate use of antimicrobials through implementation of an antimicrobial stewardship program. (Quality of evidence: MODERATE)
2	Implement diagnostic stewardship practices for ensuring appropriate use and interpretation of *C. difficile* testing. (Quality of evidence: LOW)
3	Use contact precautions for infected patients, single-patient room preferred. (Quality of evidence: LOW for hand hygiene; MODERATE for gloves; LOW for gowns; LOW for single-patient room)
4	Adequately clean and disinfect equipment and the environment of patients with CDI. (Quality of evidence: LOW for equipment; LOW for environment)
5	Assess the adequacy of room cleaning. (Quality of evidence: LOW)
6	Implement a laboratory-based alert system to provide immediate notification to infection preventionists and clinical personnel about newly diagnosed patients with CDI. (Quality of evidence: LOW)
7	Conduct CDI surveillance and analyze and report CDI data. (Quality of evidence: LOW)
8	Educate healthcare personnel (HCP), environmental service personnel, and hospital administration about CDI. (Quality of evidence: LOW)
9	Educate patients and their families about CDI as appropriate. (Quality of evidence: LOW)
10	Measure compliance with CDC or WHO hand hygiene and contact precaution recommendations. (Quality of evidence: LOW)

Additional approaches	
1	Intensify the assessment of compliance with process measures. (Quality of evidence: LOW)
2	Perform hand hygiene with soap and water as the preferred method following care of or interacting with the healthcare environment of a patient with CDI. (Quality of evidence: LOW)
3	Place patients with diarrhea on contact precautions while *C. difficile* testing is pending. (Quality of evidence: LOW)
4	Prolong the duration of contact precautions after the patient becomes asymptomatic until hospital discharge. (Quality of evidence: LOW)
5	Use an EPA-approved sporicidal disinfectant, such as diluted (1:10) sodium hypochlorite, for environmental cleaning and disinfection. Implement a system to coordinate with environmental services if it is determined that sodium hypochlorite is needed for environmental disinfection. (Quality of evidence: LOW)

### Strategies to prevent methicillin-resistant Staphylococcus aureus (MRSA) transmission and infection

**Table:** 

Essential practices	
1	Implement an MRSA monitoring program. (Quality of evidence: LOW)
2	Conduct an MRSA risk assessment. (Quality of evidence: LOW)
3	Promote compliance with CDC or World Health Organization (WHO) hand hygiene recommendations. (Quality of evidence: MODERATE)
4	Use contact precautions for MRSA-colonized and MRSA-infected patients. A facility that chooses or has already chosen to modify the use of contact precautions for some or all of these patients should conduct an MRSA-specific risk assessment to evaluate the facility for transmission risks and to assess the effectiveness of other MRSA risk mitigation strategies (eg, hand hygiene, cleaning and disinfection of the environment, single occupancy patient rooms) and should establish a process for ongoing monitoring, oversight, and risk assessment. (Quality of evidence: MODERATE)
5	Ensure cleaning and disinfection of equipment and the environment. (Quality of evidence: MODERATE)
6	Implement a laboratory-based alert system that notifies healthcare personnel (HCP) of new MRSA-colonized or MRSA-infected patients in a timely manner. (Quality of evidence: LOW)
7	Implement an alert system that identifies readmitted or transferred MRSA-colonized or MRSA-infected patients. (Quality of evidence: LOW)
8	Provide MRSA data and outcome measures to key stakeholders, including senior leadership, physicians, nursing staff, and others. (Quality of evidence: LOW)
9	Educate healthcare personnel about MRSA. (Quality of evidence: LOW)
10	Educate patients and families about MRSA. (Quality of evidence: LOW)
11	Implement an antimicrobial stewardship program. (Quality of evidence: LOW)

Additional approaches	
**Active surveillance testing (AST)**	
1	Implement an MRSA AST program for select patient populations as part of a multifaceted strategy to control and prevent MRSA. (Quality of evidence: MODERATE) Note: specific populations may have different evidence ratings.
2	Active surveillance for MRSA in conjunction with decolonization can be performed in targeted populations prior to surgery to prevent postsurgical MRSA infection. (Quality of evidence: MODERATE)
3	Active surveillance with contact precautions is inferior to universal decolonization for reduction of MRSA clinical isolates in adult ICUs. (Quality of evidence: HIGH)
4	Hospital-wide active surveillance for MRSA can be used in conjunction with contact precautions to reduce the incidence of MRSA infection. (Quality of evidence: MODERATE)
5	Active surveillance can be performed in the setting of an MRSA outbreak or evidence of ongoing transmission of MRSA within a unit as part of a multifaceted strategy to halt transmission. (Quality of evidence: MODERATE)
**Screen healthcare personnel for MRSA infection or colonization**	
1	Screen HCP for MRSA infection or colonization if they are epidemiologically linked to a cluster of MRSA infections. (Quality of evidence: LOW)
**MRSA decolonization therapy**	
1	Use universal decolonization (ie, daily CHG bathing plus 5 days of nasal decolonization) for all patients in adult ICUs to reduce endemic MRSA clinical cultures. (Quality of evidence: HIGH)
2	Perform preoperative nares screening with targeted use of CHG and nasal decolonization in MRSA carriers to reduce MRSA SSI from surgical procedures involving implantation of hardware. (Quality of evidence: MODERATE)
3	Screen for MRSA and provide targeted decolonization with CHG bathing and nasal decolonization to MRSA carriers in surgical units to reduce postoperative MRSA inpatient infections. (Quality of evidence: MODERATE)
4	Provide CHG bathing plus nasal decolonization to known MRSA carriers outside the ICU with medical devices, specifically central lines, midline catheters, and lumbar drains to reduce MRSA clinical cultures. (Quality of evidence: MODERATE)
5	Consider postdischarge decolonization of MRSA carriers to reduce postdischarge MRSA infections and readmissions. (Quality of evidence: HIGH)
6	Neonatal ICUs should consider targeted or universal decolonization during times of above-average MRSA infection rates or targeted decolonization for patients at high risk of MRSA infection (eg, low birth weight, indwelling devices, or prior to high-risk surgeries). (Quality of evidence: MODERATE)
7	Burn units should consider targeted or universal decolonization during times of above-average MRSA infection rates. (Quality of evidence: MODERATE)
8	Consider targeted or universal decolonization of hemodialysis patients. (Quality of evidence: MODERATE)
9	Decolonization should be strongly considered as part of a multimodal approach to control MRSA outbreaks. (Quality of evidence: MODERATE)
**Universal use of gowns and gloves**	
1	Use gowns and gloves when providing care to or entering the room of any adult ICU patient, regardless of MRSA colonization status. (Quality of evidence: MODERATE)

### Strategies to prevent surgical-site infections (SSIs)

**Table:** 

Essential practices	
1	Administer antimicrobial prophylaxis according to evidence-based standards and guidelines. (Quality of evidence: HIGH)
2	Use a combination of parenteral and oral antimicrobial prophylaxis prior to elective colorectal surgery to reduce the risk of SSI. (Quality of evidence: HIGH)
3	Decolonize surgical patients with an anti-staphylococcal agent in the preoperative setting for orthopedic and cardiothoracic procedures. (Quality of evidence: HIGH) Decolonize surgical patients in other procedures at high risk of staphylococcal SSI, such as those involving prosthetic material. (Quality of evidence: LOW)
4	Use antiseptic-containing preoperative vaginal preparation agents for patients undergoing cesarean delivery or hysterectomy. (Quality of evidence: MODERATE)
5	Do not remove hair at the operative site unless the presence of hair will interfere with the surgical procedure. (Quality of evidence: MODERATE)
6	Use alcohol-containing preoperative skin preparatory agents in combination with an antiseptic. (Quality of evidence: HIGH)
7	For procedures not requiring hypothermia, maintain normothermia (temperature >35.5 °C) during the perioperative period. (Quality of evidence: HIGH).
8	Use impervious plastic wound protectors for gastrointestinal and biliary tract surgery. (Quality of evidence: HIGH)
9	Perform intraoperative antiseptic wound lavage. (Quality of evidence: MODERATE)
10	Control blood glucose level during the immediate postoperative period for all patients. (Quality of evidence: HIGH)
11	Use a checklist and/or bundle to ensure compliance with best practices to improve surgical patient safety. (Quality of evidence: HIGH)
12	Perform surveillance for SSI. (Quality of evidence: MODERATE)
13	Increase the efficiency of surveillance by utilizing automated data. (Quality of evidence: MODERATE)
14	Provide ongoing SSI rate feedback to surgical and perioperative personnel and leadership. (Quality of evidence: MODERATE)
15	Measure and provide feedback to healthcare personnel (HCP) regarding rates of compliance with process measures. (Quality of evidence: LOW)
16	Educate surgeons and perioperative personnel about SSI prevention measures. (Quality of evidence: LOW)
17	Educate patients and their families about SSI prevention as appropriate. (Quality of evidence: LOW)
18	Implement policies and practices to reduce the risk of SSI for patients that align with applicable evidence-based standards, rules and regulations, and medical device manufacturer instructions for use. (Quality of evidence: MODERATE)
19	Observe and review operating room personnel and the environment of care in the operating room and in central sterile reprocessing. (Quality of evidence: LOW)

Additional approaches	
1	Perform an SSI risk assessment. (Quality of evidence: LOW)
2	Consider use of negative-pressure dressings in patients who may benefit. (Quality of evidence: MODERATE)
3	Observe and review practices in the preoperative clinic, post-anesthesia care unit, surgical intensive care unit, and/or surgical ward. (Quality of evidence: MODERATE)
4	Use antiseptic-impregnated sutures as a strategy to prevent SSI. (Quality of evidence: MODERATE)

### Strategies to prevent ventilator-associated pneumonia (VAP) and ventilator-associated events (VAEs)

#### Adult patients

**Table:** 

**Essential practices**	
*Interventions with little risk of harm and that are associated with decreases in duration of mechanical ventilation, length of stay, mortality, antibiotic utilization, and/or costs*	
**Avoid intubation and prevent reintubation if possible.**	
1	Use high flow nasal oxygen or non-invasive positive pressure ventilation (NIPPV) as appropriate, whenever safe and feasible. (Quality of evidence: HIGH)
**Minimize sedation.**	
1	Minimize sedation of ventilated patients whenever possible. (Quality of evidence: HIGH)
2	Preferentially use multimodal strategies and medications other than benzodiazepines to manage agitation. (Quality of evidence: HIGH)
3	Utilize a protocol to minimize sedation. (Quality of evidence: HIGH)
4	Implement a ventilator liberation protocol. (Quality of evidence: HIGH)
**Maintain and improve physical conditioning.**	
1	Provide early exercise and mobilization. (Quality of evidence: MODERATE)
**Elevate the head of the bed to 30°–45°. (Quality of evidence: LOW)**	
**Provide oral care with toothbrushing but without chlorhexidine. (Quality of evidence: MODERATE)**	
**Provide early enteral rather than parenteral nutrition. (Quality of evidence: HIGH)**	
**Maintain ventilator circuits.**	
1	Change the ventilator circuit only if visibly soiled or malfunctioning (or per manufacturers’ instructions) (Quality of evidence: HIGH).

**Additional approaches**	
*May decrease duration of mechanical ventilation, length of stay, and/or mortality in some populations but not in others, and they may confer some risk of harm in some populations.*	
1	Consider using selective decontamination of the oropharynx and digestive tract to decrease microbial burden in ICUs with low prevalence of antibiotic resistant organisms. Antimicrobial decontamination is not recommended in countries, regions, or ICUs with high prevalence of antibiotic-resistant organisms. (Quality of evidence: HIGH)

**Additional approaches**	
*May lower VAP rates, but current data are insufficient to determine their impact on duration of mechanical ventilation, length of stay, and mortality.*	
1	Consider using endotracheal tubes with subglottic secretion drainage ports to minimize pooling of secretions above the endotracheal cuff in patients likely to require >48–72 hours of intubation. (Quality of evidence: MODERATE)
2	Consider early tracheostomy. (Quality of evidence: MODERATE)
3	Consider postpyloric feeding tube placement in patients with gastric feeding intolerance at high risk for aspiration. (Quality of evidence: MODERATE)

#### Preterm neonatal patients

**Table:** 

**Essential practices**	
*Confer minimal risk of harm and may lower VAP and/or PedVAE rates.*	
**Avoid intubation. (Quality of evidence: HIGH)**	
**Minimize duration of mechanical ventilation. (Quality of evidence: HIGH)**	
1	Manage patients without sedation whenever possible. (Quality of evidence: LOW)
2	Use caffeine therapy for apnea of prematurity within 72 hours after birth to facilitate extubation. (Quality of evidence: HIGH)
3	Assess readiness to extubate daily. (Quality of evidence: LOW)
4	Take steps to minimize unplanned extubation and reintubation. (Quality of evidence: LOW)
5	Provide regular oral care with sterile water (extrapolated from practice in infants and children, no data in preterm neonates). (Quality of evidence: LOW)
6	Change the ventilator circuit only if visibly soiled or malfunctioning or according to the manufacturer’s instructions for use (extrapolated from studies in adults and children, no data in preterm neonates). (Quality of evidence: LOW)

**Additional approaches**	
*Minimal risks of harm, but impact on VAP and VAE rates is unknown.*	
1	Lateral recumbent positioning. (Quality of evidence: LOW)
2	Reverse Trendelenberg positioning. (Quality of evidence: LOW)
3	Closed or in-line suctioning. (Quality of evidence: LOW)
4	Oral care with maternal colostrum. (Quality of evidence: MODERATE)

#### Pediatric patients

**Table:** 

**Essential practices**	
*Confer minimal risk of harm and some data suggest that they may lower VAP rates, PedVAE rates, and/or duration of mechanical ventilation.*	
**Avoid intubation.**	
1	Use noninvasive positive pressure ventilation (NIPPV) or high-flow oxygen by nasal cannula whenever safe and feasible. (Quality of evidence: MODERATE)
**Minimize duration of mechanical ventilation.**	
1	Assess readiness to extubate daily using spontaneous breathing trials in patients without contraindications. (Quality of evidence: MODERATE)
2	Take steps to minimize unplanned extubations and reintubations. (Quality of evidence: LOW)
3	Avoid fluid overload. (Quality of evidence: MODERATE)
**Provide regular oral care (ie, toothbrushing or gauze if no teeth). (Quality of evidence: LOW)**	
**Elevate the head of the bed unless medically contraindicated. (Quality of evidence: LOW)**	
**Maintain ventilator circuits.**	
1	Change ventilator circuits only when visibly soiled or malfunctioning (or per manufacturer’s instructions). (Quality of evidence: MODERATE)
2	Remove condensate from the ventilator circuit frequently and avoid draining the condensate toward the patient. (Quality of evidence: LOW)
**Endotracheal tube selection and management**	
1	Use cuffed endotracheal tubes. (Quality of evidence: LOW)
2	Maintain cuff pressure and volume at the minimal occlusive settings to prevent clinically significant air leaks around the endotracheal tube, typically 20-25cm H_2_O. This “minimal leak” approach is associated with lower rates of post-extubation stridor. (Quality of evidence: LOW)
3	Suction oral secretions before each position change. (Quality of evidence: LOW)

**Additional approaches**	
*Minimal risks of harm and some evidence of benefit in adult patients but data in pediatric populations are limited.*	
1	Minimize sedation. (Quality of evidence: MODERATE)
2	Use endotracheal tubes with subglottic secretion drainage ports for patients ≥10 years of age. (Quality of evidence: LOW)
3	Consider early tracheostomy. (Quality of evidence: LOW)

### Strategies to prevent nonventilator hospital-acquired pneumonia (NV-HAP)

**Table:** 

**Practices supported by interventional studies suggesting lower NV-HAP rates**	
1	Provide regular oral care.
2	Diagnose and manage dysphagia.
3	Provide early mobilization.
4	Implement multimodal interventions to prevent viral infections.
5	Use prevention bundles.

### Strategies to prevent healthcare-associated infections through hand hygiene

**Table:** 

Essential practices	
**Promote the maintenance of healthy hand skin and nails. (Quality of evidence: HIGH)**	
1	Promote the preferential use of alcohol-based hand sanitizer (ABHS) in most clinical situations. (Quality of evidence: HIGH)
2	Perform hand hygiene as indicated by CDC or the WHO Five Moments. (Quality of evidence: HIGH)
3	Include fingernail care in facility-specific policies related to hand hygiene. (Quality of evidence: HIGH) a) Healthcare personnel (HCP) should maintain short, natural fingernails. b) Nails should not extend past the fingertip. c) HCP who provide direct or indirect care in high-risk areas (eg, ICU or perioperative) should not wear artificial fingernail extenders. d) Prohibitions against fingernail polish (standard or gel shellac) are at the discretion of the infection prevention program, except among scrubbed individuals who interact with the sterile field during surgical procedures; these individuals should not wear fingernail polish or gel shellac.
4	Engage all HCP in primary prevention of occupational irritant and allergic contact dermatitis. (Quality of evidence: HIGH)
5	Provide cotton glove liners for HCP with hand irritation and educate these HCP on their use. (Quality of evidence: MODERATE)
**Select appropriate products.**	
1	For routine hand hygiene, choose liquid, gel, or foam ABHS with at least 60% alcohol. (Quality of evidence: HIGH)
2	Involve HCP in selection of products. (Quality of evidence: HIGH)
3	Obtain and consider manufacturers’ product-specific data if seeking ABHS with ingredients that may enhance efficacy against organisms anticipated to be less susceptible to biocides. (Quality of evidence: MODERATE)
4	Confirm that the volume of ABHS dispensed is consistent with the volume shown to be efficacious. (Quality of evidence: HIGH)
5	Educate HCP about an appropriate volume of ABHS and the time required to obtain effectiveness. (Quality of evidence: HIGH)
6	Provide facility-approved hand moisturizer that is compatible with antiseptics and gloves. (Quality of evidence: HIGH)
7	For surgical antisepsis, use an FDA-approved surgical hand scrub or waterless surgical hand rub. (Quality of evidence: HIGH)
**Ensure the accessibility of hand hygiene supplies. (Quality of evidence: HIGH)**	
1	Ensure ABHS dispensers are unambiguous, visible, and accessible within the workflow of HCP. (Quality of evidence: HIGH)
2	In private rooms, consider 2 ABHS dispensers the minimum threshold for adequate numbers of dispensers: 1 dispenser in the hallway, and 1 in the patient room. (Quality of evidence: HIGH)
3	In semiprivate rooms, suites, bays, and other multipatient bed configurations, consider 1 dispenser per 2 beds the minimum threshold for adequate numbers of dispensers. Place ABHS dispensers in the workflow of HCP. (Quality of evidence: LOW)
4	Ensure that the placement of hand hygiene supplies (eg, individual pocket-sized dispensers, bed mounted ABHS dispenser, single use pump bottles) is easily accessible for HCP in all areas where patients receive care. (Quality of evidence: HIGH)
5	Evaluate for the risk of intentional consumption. Utilize dispensers that mitigate this risk, such as wall-mounted dispensers that allow limited numbers of activations within short periods (eg, 5 seconds). (Quality of evidence: LOW)
6	Have surgical hand rub and scrub available in perioperative areas. (Quality of evidence: HIGH)
7	Consider providing ABHS hand rubs or handwash with FDA-approved antiseptics for use in procedural areas and prior to high-risk bedside procedures (eg, central-line insertion). (Quality of evidence: LOW)
**Ensure appropriate glove use to reduce hand and environmental contamination. (Quality of Evidence: HIGH)**	
1	Use gloves for all contact with the patient and environment as indicated by standard and contact precautions during the care of individuals with organisms confirmed to be less susceptible to biocides (e.g., *C. difficile* or norovirus)
2	Educate HCP about the potential for self-contamination and environmental contamination when gloves are worn. (Quality of evidence: HIGH)
3	Educate and confirm the ability of HCP to doff gloves in a manner that avoids contamination. (Quality of evidence: HIGH)
**Take steps to reduce environmental contamination associated with sinks and sink drains. (Quality of evidence: HIGH)**	
**Monitor adherence to hand hygiene. (Quality of evidence: HIGH)**	
**Provide timely and meaningful feedback to enhance a culture of safety. (Quality of evidence: MODERATE)**	

Additional approaches during outbreaks	
1	Consider educating HCP using a structured approach (eg, WHO Steps) for handwashing or hand sanitizing. Evaluate HCP adherence to technique. (Quality of evidence: LOW)
2	For waterborne pathogens of premise plumbing, consider disinfection of sink drains using an EPA-registered disinfectant with claims against biofilms. Consult with state or local public health for assistance in determining appropriate protocols for use and other actions needed to ensure safe supply. (Quality of evidence: LOW)
3	For *C. difficile* and norovirus, in addition to contact precautions, encourage hand washing with soap and water after the care of patients with known or suspected infections. (Quality of evidence: LOW)

### Implementing strategies to prevent healthcare-associated infections

#### Standard approach to implementation

**Table:** 

1	Assess determinants of change and classify as follows: • **Facilitators**: promote practice or change, or • **Barriers**: hinder practice or change	**Individual level**: healthcare personnel, leaders, patients, and visitors’ preferences, needs, attitudes, and knowledge. **Facility level**: team composition, communication, culture, capacity, policies, resources. **Partners**: degree of support and buy-in.
2	Choose measures	Measurement methods must be appropriate for the question(s) they seek to answer and adhere to the methods’ data collection and analysis rules: • **Outcome measure**: ultimate goal (eg, HAI reduction). • **Process measure**: action reliability (eg, bundle adherence). • **Balancing measure**: undesired outcome of change (eg, staff absences due to required vaccine side effects).
3	Select framework(s)	See below and “Implementing Strategies to Prevent Infections in Acute Care Settings” (Table 3)^32^

#### Examples of implementation frameworks

**Table:** 

Framework	Published Experience	Resources
4Es	Settings • Healthcare facilities • Large-scale projects including multiple sites Infection prevention and control • HAI prevention (including mortality reduction and cost savings)	• 4Es Framework^11^ • HAI reduction^12–14^ • Mortality reduction^15^ • Cost savings^16^
Behavior Change Wheel	Settings • Community-based practice • Healthcare facilities Healthy behaviors • Smoking cessation • Obesity prevention • Increased physical activity Infection prevention and control • Hand hygiene adherence • Antibiotic prescribing^17^	• Behavior Change Wheel: A Guide to Designing Interventions^18^ • Stand More at Work (SMArT Work)^19^
Comprehensive Unit-based Safety Program (CUSP)	Settings • Intensive care units • Ambulatory centers Improvements • Antibiotic prescribing • CLABSI prevention • CAUTI prevention	• CUSP Implementation Toolkit^20^ • AHA/HRET: Eliminating CAUTI (Stop CAUTI)^21^ • AHRQ Toolkit to Improve Safety in Ambulatory Surgery Centers^22^
European Mixed Methods	Settings • European institutions of varied healthcare systems and cultures Improvements: • CLABSI prevention • Hand hygiene	• PROHIBIT: Description and Materials^23^
Getting to Outcomes (GTO)®	Settings • Community programs and services Improvements • Sexual health promotion • Dual-disorder treatment program in veterans • Community emergency preparedness	• RAND Guide for Emergency Preparedness^24^ (illustrated overview of GTO® methodology)
Model for Improvement	Settings • Healthcare (inpatient, perioperative, ambulatory) • Public health Interventions • PPE use • HAI prevention • Public health process evaluation	• Institute for Healthcare Improvement^25^ • The Improvement Guide^26^ • Deming’s System of Profound Knowledge^27^
Reach, Effectiveness, Adoption, Implementation, Maintenance (RE-AIM)	Settings • Healthcare • Public health • Community programs • Sexual health Evaluations • Antimicrobial stewardship in the ICU • Clinical practice guidelines for STIs • Promotion of vaccination • Implementation of contact tracing	• RE-AIM.org^28^ • Understanding and applying the RE-AIM framework: Clarifications and resources^29^
Replicating Effective Practices (REP)	Settings • Healthcare • Public health • HIV prevention Interventions that have produced positive results are reframed for local relevance	CDC Compendium of HIV Prevention Interventions with Evidence of Effectiveness^30^ (see Section C, Intervention Checklist)
Theoretical Domains	Settings • Healthcare (inpatient, perioperative, ambulatory) • Community (individual and community-based behaviors) Health maintenance • Diabetes management in primary care • Pregnancy weight management HCP practice • ICU blood transfusion • Selective GI tract decontamination • Preoperative testing • Spine imaging • Hand hygiene	• A guide to using the Theoretical Domains Framework of behavior change to investigate implementation problems^31^ • Developing theory-informed behavior-change interventions to implement evidence into practice: a systematic approach using the Theoretical Domains Framework^32^ • Choosing Wisely Deimplementation Framework^33^

## Appendix

**Table 1.** Quality of Evidence

HIGH	Highly confident that the true effect lies close to that of the estimated size and direction of the effect. Evidence is rated as “high” quality when there are a wide range of studies with no major limitations, there is little variation between studies, and the summary estimate has a narrow confidence interval.
MODERATE	The true effect is likely to be close to the estimated size and direction of the effect, but it is possible that it is substantially different. Evidence is rated as “moderate” quality when there are only a few studies and some have limitations but not major flaws, there is some variation between studies, or the confidence interval of the summary estimate is wide.
LOW	The true effect may be substantially different from the estimated size and direction of the effect. Evidence is rated as “low” quality when supporting studies have major flaws, there is important variation between studies, the confidence interval of the summary estimate is very wide, or there are no rigorous studies.

Based on the CDC Healthcare Infection Control Practices Advisory Committee (HICPAC) “Update to the Centers for Disease Control and Prevention and the Healthcare Infection Control Practices Advisory Committee Recommendations Categorization Scheme for Infection Control and Prevention Guideline Recommendations” (October 2019),^34^ the Grades of Recommendation, Assessment, Development, and Evaluation (GRADE),^35^ and the Canadian Task Force on Preventive Health Care.^36^

**Table 2.** Level of Recommendation

**Level of Recommendation**	**Evidence Level**	**Implied Obligation**
**Essential practice**: Panel members are confident the benefits of the recommended approach clearly exceed the harms (or, in the case of a negative recommendation, that the harms clearly exceed the benefits).	In general, high to moderate-quality evidence (Table 1) OR lesser evidence or expert opinion when high-quality evidence is not available and the anticipated benefits strongly outweigh the harms.	In general, healthcare personnel and facilities “should” implement the recommended approach unless a clear and compelling rationale for an alternative approach is present.
**Additional approach**: Panel members have determined that the benefits of the recommended approach are likely to exceed the harms (or, in the case of a negative recommendation, that the harms are likely to exceed the benefits).	• In general, may be supported by either low-, moderate-, or high-quality evidence. • There is high-quality evidence, but the benefit–harm balance is not clearly tipped in one direction. • The evidence is weak enough to cast doubt on whether the recommendation will consistently lead to benefit. • The likelihood of benefit for a specific patient population or clinical situation is extrapolated from relatively high-quality evidence demonstrating impact on other patient populations or in other clinical situations (eg, evidence obtained during outbreaks used to support probable benefit during endemic periods). • The impact of the specific intervention is difficult to disentangle from the impact of other simultaneously implemented interventions (eg, studies evaluating “bundled” practices). • There appears to be benefit based on available evidence, but the benefit/harm balance may change with further research. • Benefit is most likely if the intervention is used as a supplemental measure in addition to essential practices.	Healthcare personnel/facilities “could” or “may consider” implementing the recommended approach. The degree of appropriateness may vary depending on the benefit–harm balance for the specific setting.
**Unresolved issue**: Panel members agree that there is both a lack of pertinent evidence and unclear balance between benefits and harms.		

**Table 3.** Compendium Leadership and Authors

Society for Healthcare Epidemiology of America (SHEA) co-chair	Deborah S. Yokoe MD, MPH
Infectious Disease Society of America (IDSA) co-chair	Lisa L. Maragakis MD, MPH
Compendium Partners	The Society for Healthcare Epidemiology of America (SHEA) The Infectious Diseases Society of America (IDSA) The Association for Professionals in Infection Control and Epidemiology (APIC) The American Hospital Association (AHA) The Joint Commission
Compendium Advisory Group	Valerie M. Deloney MBA (SHEA) Kristy Weinshel MBA (SHEA) Emily Sickbert-Bennett PhD, MS, CIC (SHEA) Genet Demisashi (IDSA) Shanina Knighton PhD, RN, CIC (APIC, 2022–2023) Silvia Quevedo MS (APIC, 2018–2022) Kavita Bhat MD, MPH, CPHQ (AHA, 2023) Nancy Foster (AHA, 2018–2022) Margaret VanAmringe MHS (The Joint Commission) David Classen MD, MS (2008 IDSA Compendium co-chair)
Compendium Expert Panel	Hilary M. Babcock MD, MPH Michael Bell MD Kristina A. Bryant MD Neil O. Fishman MD Mary K. Hayden MD Elaine L. Larson PhD, RN, CIC Anurag N. Malani MD Trish M. Perl MD, MSc Edward Septimus MD Robert A. Weinstein MD
Compendium Lead Authors	Payal K. Patel MD, MPH (CAUTI) Jennifer Meddings MD, MSc (CAUTI) Sonali D. Advani MBBS, MPH (CAUTI) Niccolò Buetti MD, MSc, PhD (CLABSI) Jonas Marschall MD, MSc (CLABSI) Leonard A. Mermel DO, ScM (CLABSI) Larry K. Kociolek MD, MSCI (*C. difficile* infection) Erik R. Dubberke MD, MSPH (*C. difficile* infection) Dale N. Gerding MD (*C. difficile* infection) Janet Glowicz PhD, RN, CIC (Hand Hygiene) Katherine D. Ellingson PhD (Hand Hygiene) Emily Landon MD (Hand Hygiene) Kyle J. Popovich MD, MS (MRSA) David P. Calfee MD, MS (MRSA) Michael S. Calderwood MD, MPH (SSI) Deverick J. Anderson MD, MPH (SSI) Keith S. Kaye MD, MPH (SSI) Michael Klompas MD, MPH (VAP, VAE, NV-HAP) Sean M. Berenholtz MD, MHS (VAP, VAE, NV-HAP) Kavita K. Trivedi MD (Implementation) Joshua K. Schaffzin MD, PhD (Implementation) Sean M. Berenholtz MD, MHS (Implementation)
CAUTI Authors	Payal K. Patel MD, MPH Sonali D. Advani MBBS, MPH Aaron D. Kofman MD Evelyn Lo MD Lisa L. Maragakis MD, MPH David A. Pegues MD Ann Marie Pettis RN, BSN Sanjay Saint MD, MPH Barbara Trautner MD, PhD Deborah S. Yokoe MD, MPH Jennifer Meddings MD, MSc
CLABSI Authors	Niccolò Buetti MD, MSc, PhD Jonas Marschall MD, MSc Marci Drees MD, MS Mohamad G. Fakih MD, MPH Lynn Hadaway MEd, RN, NPD-BC, CRNI Lisa L. Maragakis MD, MPH Elizabeth Monsees PhD, MBA, RN, CIC Shannon Novosad MD, MPH Naomi P. O’Grady MD Mark E. Rupp MD Joshua Wolf MBBS, PhD Deborah S. Yokoe MD, MPH Leonard A. Mermel DO, ScM
CDI Authors	Larry K. Kociolek MD, MSCI Dale N. Gerding MD Ruth Carrico PhD, DNP, APRN, CIC Philip Carling MD Curtis J. Donskey MD Ghinwa Dumyati MD David T. Kuhar MD Vivian G. Loo MD, MSc Lisa L. Maragakis MD, MPH Monika Pogorzelska-Maziarz PhD, MPH Thomas J. Sandora MD, MPH David J. Weber MD, MPH Deborah S. Yokoe MD, MPH Erik R. Dubberke MD, MSPH
MRSA Authors	Kyle J. Popovich MD, MS Kathy Aureden MS, MT, CIC D. Cal Ham MD, MPH Anthony D. Harris MD, MPH Amanda J. Hessels PhD, MPH, RN, CIC Susan S. Huang MD, MPH Lisa L. Maragakis MD, MPH Aaron M. Milstone MD, MHS Julia Moody MS Deborah S. Yokoe MD, MPH David P. Calfee MD, MS
SSI Authors	Michael S. Calderwood MD, MPH Deverick J. Anderson MD, MPH Dale W. Bratzler DO, MPH E. Patchen Dellinger MD Sylvia Garcia-Houchins MBA, RN, CIC Lisa L. Maragakis MD, MPH Ann-Christine Nyquist MD, MSPH Kiran M. Perkins MD, MPH Michael Anne Preas RN, MS, CIC Lisa Saiman MD, MPH Joshua K. Schaffzin MD, PhD Marin Schweizer PhD Deborah S. Yokoe MD, MPH Keith S. Kaye MD, MPH
VAP, VAE, NV-HAP Authors	Michael Klompas MD, MPH Richard Branson MSc, RRT Kelly Cawcutt MD, MS Matthew Crist MD Eric C. Eichenwald MD Linda R. Greene RN, MPS, CIC Grace Lee MD Lisa L. Maragakis MD, MPH Krista Powell MD Gregory P. Priebe MD Kathleen Speck MPH Deborah S. Yokoe MD, MPH Sean M. Berenholtz MD, MHS
Hand Hygiene Authors	Janet Glowicz PhD, RN, CIC Emily Landon MD Emily E. Sickbert-Bennett PhD, MS, CIC Allison E. Aiello PhD Karen deKay MSN, RN, CNOR, CIC Karen K. Hoffmann BSN, MS, CIC Lisa L. Maragakis MD, MPH Russell Olmsted MPH, CIC Philip M. Polgreen MD, MPH Polly A. Trexler MS, CIC Margaret A. VanAmringe MHS Amber R. Wood MSN, RN, CNOR, CIC Deborah S. Yokoe MD, MPH Katherine D. Ellingson PhD
Implementation Authors	Kavita K. Trivedi MD Joshua K. Schaffzin MD, PhD Valerie M. Deloney MBA Kathy Aureden MS, MT, CIC Ruth Carrico PhD, DNP, APRN, CIC Sylvia Garcia-Houchins MBA, RN, CIC J. Hudson Garrett Jr PhD, MSN, MPH, MBA, CIC Janet Glowicz PhD, RN, CIC Grace M. Lee MD, MPH Lisa L. Maragakis MD, MPH Julia Moody MS, CIC Ann Marie Pettis RN, BSN, CIC Sanjay Saint MD, MPH Marin L. Schweizer PhD Deborah S. Yokoe MD, MPH Sean M. Berenholtz MD, MHS
Endorsing Organizations *These organizations formally reviewed and endorsed the articles that compose the *Compendium: 2022 Updates*.*	The Society for Healthcare Epidemiology of America (SHEA) The Infectious Diseases Society of America (IDSA) The Association for Professionals in Infection Control and Epidemiology (APIC) The American Hospital Association (AHA) The Joint Commission American Society of Health System Pharmacists (ASHP) for Strategies to Prevent CLABSI, VAP/VAE/NV-HAP, Hand Hygiene, CDI, SSI, and Implementation Anesthesia Patient Safety Foundation (APSF) National Foundation for Infectious Diseases (NFID) for Strategies to Prevent MRSA, CAUTI, SSI, and CDI Pediatric Infectious Diseases Society (PIDS) Society of Infectious Diseases Pharmacists (SIDP) for VAP/VAE/NV-HAP and Implementation Surgical Infection Society (SIS)
Supporting Organizations *These organizations provided general, non-financial support for the *Compendium: 2022 Updates*.*	The Council of State and Territorial Epidemiologists (CSTE) The National Association of County and City Health Officials (NACCHO) Society of Critical Care Medicine (SCCM) Society of Infectious Diseases Pharmacists (SIDP)
Organizations that endorsed or provided support for the *Compendium: 2022 Updates* after the publication of this paper are listed on the SHEA website (www.shea-online.org).	

## References

[1] Buetti N , Marschall J , Drees M , et al. Strategies to prevent central-line–associated bloodstream infections in acute-care hospitals: 2022 update. Infect Control Hosp Epidemiol 2022;43:553–569. 35437133 10.1017/ice.2022.87 PMC9096710

[2] Klompas M , Branson R , Cawcutt K , et al. Strategies to prevent ventilator-associated pneumonia, ventilator-associated events, and nonventilator hospital-acquired pneumonia in acute-care hospitals: 2022 update. Infect Control Hosp Epidemiol 2022;43:687–713. 35589091 10.1017/ice.2022.88 PMC10903147

[3] Glowicz J , Landon E , Sickbert Bennett EE , et al. Compendium of strategies to prevent healthcare-associated infections through hand hygiene: 2022 update. Infect Control Hosp Epidemiol 2023;44:355–376. 36751708 10.1017/ice.2022.304 PMC10015275

[4] Kociolek LK , Gerding DN , Carrico R , et al. Strategies to prevent *Clostridioides difficile* infections in acute-care hospitals: 2022 update. Infect Control Hosp Epidemiol 2023;44:527–549. 37042243 10.1017/ice.2023.18 PMC10917144

[5] Calderwood MS , Anderson DJ , Bratzler DW , et al. Strategies to prevent surgical-site infections in acute-care hospitals: 2022 update. Infect Control Hosp Epidemiol 2023;44:695–720. 37137483 10.1017/ice.2023.67 PMC10867741

[6] Popovich KJ , Aureden K , Ham DC , et al. SHEA/IDSA/APIC practice recommendation: strategies to prevent methicillin-resistant *Staphylococcus aureus* transmission and infection in acute-care hospitals: 2022 update. *Infect Control Hosp Epidemiol* 2023:1–29. 10.1017/ice.2023.102 PMC10369222 37381690

[7] Trivedi KK , Schaffzin J , Deloney V , et al. Implementing strategies to prevent infections in acute-care settings. *Infect Control Hosp Epidemiol* 2023:1–15. 10.1017/ice.2023.103 PMC10527889 37431239

[8] Patel P , Advani S , Kofman A , et al. Strategies to prevent catheter-associated urinary tract infections in acute care hospitals: 2022 update. *Infect Control Hosp Epidemiol* 2023. doi: 10.1017/ice.2023.158 PMC11287483 37620117

[9] Yokoe D , Maragakis L , Advani S , et al. Introduction to a compendium of strategies to prevent healthcare-associated infections in acute-care hospitals: 2022 updates. *Infect Control Hosp Epidemiol* 2023. doi: 10.1017/ice.2023.137 PMC10587365 37855077

[10] Yokoe DS , Anderson DJ , Berenholtz SM , et al. Introduction to “A compendium of strategies to prevent healthcare-associated infections in acute care hospitals: 2014 updates”. Infect Control Hosp Epidemiol 2014;35(Suppl 2):S1–S5. 10.1086/678903 25264563

[11] Pronovost PJ , Berenholtz SM , Needham DM. Translating evidence into practice: a model for large scale knowledge translation. BMJ 2008;337:a1714. 18838424 10.1136/bmj.a1714

[12] Berenholtz SM , Lubomski LH , Weeks K , et al. Eliminating central-line–associated bloodstream infections: a national patient safety imperative. Infect Control Hosp Epidemiol 2014;35:56–62. 24334799 10.1086/674384

[13] Pronovost P , Needham D , Berenholtz S , et al. An intervention to decrease catheter-related bloodstream infections in the ICU. N Engl J Med 2006;355:2725–2732. 17192537 10.1056/NEJMoa061115

[14] Pronovost PJ , Watson SR , Goeschel CA , Hyzy RC , Berenholtz SM. Sustaining reductions in central-line–associated bloodstream infections in Michigan intensive care units: a 10-year analysis. Am J Med Qual 2016;31:197–202. 25609646 10.1177/1062860614568647

[15] Lipitz-Snyderman A , Steinwachs D , Needham DM , Colantuoni E , Morlock LL , Pronovost PJ. Impact of a statewide intensive care unit quality improvement initiative on hospital mortality and length of stay: retrospective comparative analysis. BMJ 2011;342:d219. 21282262 10.1136/bmj.d219 PMC3031651

[16] Waters HR , Korn R Jr , Colantuoni E , et al. The business case for quality: economic analysis of the Michigan Keystone Patient Safety Program in ICUs. Am J Med Qual 2011;26:333–339. 21856956 10.1177/1062860611410685

[17] Rawson TM , Moore LS , Tivey AM , et al. Behaviour change interventions to influence antimicrobial prescribing: a cross-sectional analysis of reports from UK state-of-the-art scientific conferences. Antimicrob Resist Infect Control 2017;6:11. 28101333 10.1186/s13756-017-0170-7 PMC5237267

[18] Michie S , Atkins L , West R. The Behavior Change Wheel: A Guide to Designing Interventions. Sutton, England: Silverback Publishing; 2014.

[19] Munir F , Biddle SJH , Davies MJ , et al. Stand More AT Work (SMArT Work): using the behaviour change wheel to develop an intervention to reduce sitting time in the workplace. BMC Public Health 2018;18:319. 29510715 10.1186/s12889-018-5187-1 PMC5840779

[20] How to Use the CUSP Toolkit. Agency for Healthcare Research and Quality website. https://www.ahrq.gov/hai/cusp/toolkit/index.html. Updated July 2018. Accessed July 12, 2023.

[21] Eliminating catheter-associated urinary tract infections. American Hospital Association website. http://www.hpoe.org/resources/ahahret-guides/1420. Accessed July 12, 2023.

[22] Toolkit to Improve Safety in Ambulatory Surgery Centers. Agency for Healthcare Research and Quality website. https://www.ahrq.gov/hai/tools/ambulatory-surgery/index.html. Updated 2023. Accessed July 12, 2023.

[23] van der Kooi TII , Smid EA , Koek MBG , et al. The effect of an intervention bundle to prevent central venous catheter–related bloodstream infection in a national programme in the Netherlands. J Hosp Infect 2023;131:194–202. 36414165 10.1016/j.jhin.2022.11.006

[24] Ebener P , Hunter S , Adams R , Eisenman D , Acosta J , Chinman M. Getting to outcomes guide for community emergency preparedness. RAND Corporation website. https://www.rand.org/pubs/tools/TL259.html. Published 2017. Accessed July 12, 2023.

[25] Resources. Institute for Healthcare Improvement website. https://www.ihi.org/resources/Pages/default.aspx. Accessed July 12, 2023.

[26] Langley G , Moen R , Nolan KM , Nolan T , Norman C , Provost L. The improvement guide: a practical approach to enhancing organizational performance. Agency for Healthcare Research and Quality website. https://psnet.ahrq.gov/issue/improvement-guide-practical-approach-enhancing-organizational-performance-2nd-ed. Accessed July 12, 2023.

[27] The Deming System of Profound Knowledge (SoPK). The W. Edwards Deming Institute website. https://deming.org/explore/sopk/

[28] RE-AIM. RE-AIM and PRISM: implementation in context. https://re-aim.org/

[29] Holtrop JS , Estabrooks PA , Gaglio B , et al. Understanding and applying the RE-AIM framework: clarifications and resources. J Clin Transl Sci 2021;5:e126. 34367671 10.1017/cts.2021.789 PMC8327549

[30] HIV/AIDS Prevention Research Synthesis Project. Compendium of HIV prevention interventions with evidence of effectiveness. Centers for Disease Control and Prevention website. https://www.cdc.gov/hiv/pdf/research/interventionresearch/rep/prevention_research_compendium.pdf. Published November 1999. Revised August 2001. Accessed July 2023.

[31] Atkins L , Francis J , Islam R , et al. A guide to using the theoretical domains framework of behaviour change to investigate implementation problems. Implement Sci 2017;12:77. 28637486 10.1186/s13012-017-0605-9 PMC5480145

[32] French SD , Green SE , O’Connor DA , et al. Developing theory-informed behaviour change interventions to implement evidence into practice: a systematic approach using the theoretical domains framework. Implement Sci 2012;7:38. 22531013 10.1186/1748-5908-7-38 PMC3443064

[33] Grimshaw JM , Patey AM , Kirkham KR , et al. De-implementing wisely: developing the evidence base to reduce low-value care. BMJ Qual Saf 2020;29:409–417. 10.1136/bmjqs-2019-010060 PMC7229903 32029572

[34] Update to the CDC and the HICPAC Recommendation Categorization Scheme for Infection Control and Prevention Guideline Recommendations. Centers for Disease Control and Prevention website. https://www.cdc.gov/hicpac/workgroup/recommendation-scheme-update.html. Accessed July 12, 2023.

[35] Guyatt GH , Oxman AD , Vist GE , et al. GRADE: an emerging consensus on rating quality of evidence and strength of recommendations. BMJ 2008;336:924–926. 18436948 10.1136/bmj.39489.470347.AD PMC2335261

[36] Grades of Recommendation, Assessment, Development, and Evaluation (GRADE). Canadian Task Force on Preventive Health Care website. https://canadiantaskforce.ca/wp-content/uploads/2018/12/GRADE-Info-for-website-blue-format-181220.pdf. Accessed July 12, 2023.

